# Hyperspectral estimation of chlorophyll density in winter wheat using fractional-order derivative combined with machine learning

**DOI:** 10.3389/fpls.2024.1492059

**Published:** 2025-01-14

**Authors:** Chenbo Yang, Meichen Feng, Juan Bai, Hui Sun, Rutian Bi, Lifang Song, Chao Wang, Yu Zhao, Wude Yang, Lujie Xiao, Meijun Zhang, Xiaoyan Song

**Affiliations:** ^1^ College of Agriculture, Shanxi Agricultural University, Taigu, Shanxi, China; ^2^ College of Resources and Environment, Shanxi Agricultural University, Taigu, Shanxi, China; ^3^ Life Sciences Department, Yuncheng University, Yuncheng, Shanxi, China

**Keywords:** hyperspectral, chlorophyll density, fractional-order derivative, competitive adaptive reweighted sampling, machine learning

## Abstract

Chlorophyll density (ChD) can reflect the photosynthetic capacity of the winter wheat population, therefore achieving real-time non-destructive monitoring of ChD in winter wheat is of great significance for evaluating the growth status of winter wheat. Derivative preprocessing has a wide range of applications in the hyperspectral monitoring of winter wheat chlorophyll. In order to research the role of fractional-order derivative (FOD) in the hyperspectral monitoring model of ChD, this study based on an irrigation experiment of winter wheat to obtain ChD and canopy hyperspectral reflectance. The original spectral reflectance curves were preprocessed using 3 FOD methods: Grünwald-Letnikov (GL), Riemann-Liouville (RL), and Caputo. Hyperspectral monitoring models for winter wheat ChD were constructed using 8 machine learning algorithms, including partial least squares regression, support vector regression, multi-layer perceptron regression, random forest regression, extra-trees regression (ETsR), decision tree regression, K-nearest neighbors regression, and gaussian process regression, based on the full spectrum band and the band selected by competitive adaptive reweighted sampling (CARS). The main results were as follows: For the 3 types of FOD, GL-FOD was suitable for analyzing the change process of the original spectral curve towards the integer-order derivative spectral curve. RL-FOD was suitable for constructing the hyperspectral monitoring model of winter wheat ChD. Caputo-FOD was not suitable for hyperspectral research due to its insensitivity to changes in order. The 3 FOD calculation methods could all improve the correlation between the original spectral curve and Log(ChD) to varying degrees, but only the GL method and RL method could observe the change process of correlation with order changes, and the shorter the wavelength, the smaller the order, and the higher the correlation. The bands screened by CARS were distributed throughout the entire spectral range, but there was a relatively concentrated distribution in the visible light region. Among all models, CARS was used to screen bands based on the 0.3-order RL-FOD spectrum, and the model constructed using ETsR reached the best accuracy and stability. Its R^2c^, RMSE_c_, R^2v^, RMSE_v_, and RPD were 1.0000, 0.0000, 0.8667, 0.1732, and 2.6660, respectively. In conclusion, based on the winter wheat ChD data set and the corresponding canopy hyperspectral data set, combined with 3 FOD calculation methods, 1 band screening method, and 8 modeling algorithms, this study constructed hyperspectral monitoring models for winter wheat ChD. The results showed that based on the 0.3-order RL-FOD, combined with the CARS screening band, ETsR modeling has the highest accuracy, and hyperspectral estimation of winter wheat ChD can be realized. The results of this study can provide some reference for the rapid and nondestructive estimation of ChD in winter wheat.

## 1 Introduction

Chlorophyll is an important pigment for photosynthesis in most plants, so it plays a vital role in the growth and development of plants (Xu et al., 2001; Chatterjee and Kundu, 2015). Wheat is an important food crop, and about one-third of the world’s population eats wheat as a staple food (Jiang et al., 2021). However, during its growth process, it is easily affected by factors such as water, fertilizer, and diseases, which hinder the synthesis of chlorophyll, thereby affecting photosynthesis and leading to reduced yield (He et al., 2018; Yang et al., 2021). Chlorophyll density (ChD) is an indicator that can evaluate the chlorophyll content of crop populations. During the growth process of wheat, obtaining ChD can be used to evaluate the overall growth status of wheat and provide a reference for adjusting agricultural production measures (Liu et al., 2017; Xie et al., 2020). However, traditional methods for obtaining ChD commonly suffer from issues such as destructive sampling, complex measurement processes, and unable to obtain data in real-time. Therefore, there is an urgent need for a fast, real-time, and non-destructive method to obtain wheat ChD to meet the current needs of precision agriculture development.

Remote sensing technology has the advantage of quick and non-destructive acquisition of target object features, providing technical support for real-time acquisition of crop growth status. Especially hyperspectral remote sensing technology, with its advantages of high resolution and large spectral information, has been used by many scholars to quantitatively monitor wheat chlorophyll. For example, Li et al. (2022); Zhang et al. (2022), and Jiang et al. (2010) all performed 1.0-order derivative preprocessing on the original spectral curve, and used multiple vegetation indices combined with linear, exponential, and power regression models to monitor wheat chlorophyll content and ChD, respectively, all achieved good monitoring results. Huang et al. (2010) performed multiple scattering correction, 1.0-order derivative, and 2.0-order derivative preprocessing on spectral curve, and constructed monitoring models of wheat chlorophyll content using partial least squares regression (PLSR) and stepwise multiple linear regression. The results showed that the stepwise multiple linear regression model combining multiple scattering correction with 2.0-order derivative reached the best performance. It can be seen that previous scholars have conducted relatively mature research on quantitative monitoring of wheat chlorophyll using hyperspectral technology.

Through analysis of previous research, it can be seen that in the process of constructing hyperspectral monitoring models of winter wheat chlorophyll content or ChD, previous scholars preprocessed the original spectral curve to varying degrees, such as derivatives, to reduce noise interference, and to reduce model complexity by constructing vegetation indices. Finally, by comparing the accuracy of various modeling methods, to achieve rapid estimation of winter wheat chlorophyll. It can be seen that appropriate preprocessing, bands, and modeling algorithms are all important means to improve the accuracy of hyperspectral models.

For preprocessing, derivative preprocessing was widely used in hyperspectral studies of other crops and growth physiological parameters, and was considered to have good effects (Ji et al., 2022; Liu et al., 2022; Yang et al., 2023b). However, these studies all used integer-order derivatives, but there is a significant difference between the original spectral curve and the integer-order derivative spectral curve. This means that there may be curves with higher signal-to-noise ratios during the transition from the original spectral curve to the integer-order derivative curve. In order to find this curve, some studies used FOD to further analyze the role of derivative preprocessing in hyperspectral monitoring models of crop growth physiological parameters. For example, Li et al (2021a; 2021b; 2023). applied FOD to hyperspectral monitoring of wheat leaf moisture content, leaf area index, and chlorophyll content, Bhadra et al. (2020) applied FOD to hyperspectral monitoring of sorghum ChD, all achieved good monitoring results. FOD is a mathematical concept that can be defined in many ways, with Grünwald-Letnikov (GL), Riemann Liouville (RL), and Caputo being the most widely used definitions (Benkhettou et al., 2014). Among them, GL method is defined based on discrete points. Both RL method and Caputo method calculate derivatives by curve fitting discrete points first. But when calculating the traditional integer-order derivative, the curve fitting is performed first and then the derivative is calculated. However, in the preprocessing of spectral curves with FOD, most of the predecessors used GL method. The reason is that the data constituting the spectral curve itself is discrete point data, which is more consistent with the definition of GL method. This leads to a certain difference between its calculation of integer-order derivative and traditional methods. Therefore, in order to more accurately observe the role of different orders of FOD preprocessing in the construction of winter wheat ChD, it is necessary to compare the three calculation methods at the same time.

For band selection, many studies chosen to reduce model complexity by constructing vegetation index when constructing hyperspectral monitoring models of winter wheat ChD (Zhao et al., 2011; Xing et al., 2022). However, the vegetation index often only retains a few bands in the full spectrum band, which may result in the loss of much useful spectral information in the spectral curve. Therefore, it is necessary to use appropriate band selection algorithms to screen an appropriate number of band to improve the utilization rate of spectral information (Wang et al., 2022). Competitive adaptive reweighted sampling (CARS) is a screening algorithm based on the regression coefficients of the PLSR model, which has a wide range of applications in hyperspectral model research (Chen et al., 2023; Li and Yang, 2023). This study will use CARS to screen band that are important to ChD.

For modeling algorithms, a large number of current studies have shown that using machine learning algorithms to construct hyperspectral monitoring models has become one of the hot directions in the field of hyperspectral research, and there have been many reports on wheat chlorophyll (Sun et al., 2007; Wang et al., 2022; Feng et al., 2023; Huang et al., 2023). Based on previous research, it can be seen that PLSR, support vector regression (SVR), multi-layer perceptron regression (MLPR), random forest regression (RFR), extra-trees regression (ETsR), decision tree regression (DTR), K-nearest neighbors regression (KNR), and gaussian process regression (GPR) are commonly used machine learning algorithms. This study will use these algorithms to construct hyperspectral monitoring models.

Based on the above analysis, this study will use winter wheat as the research material, preprocess the original spectral curve using GL, RL, and Caputo FOD calculation methods, screen the sensitive band of winter wheat ChD using CARS method, and construct hyperspectral monitoring models of winter wheat ChD using various modeling methods such as PLSR, SVR, MLPR, RFR, ETsR, DTR, KNR, and GPR. Hope to achieve hyperspectral monitoring of ChD in winter wheat. The aims of this study are: (1) Clarify the preprocessing effects of GL, RL, and Caputo FOD calculation methods on the original spectral curve and their relationship with winter wheat ChD; (2) Analyze the effects of CARS method in screening ChD sensitive band in winter wheat; (3) Under 3 types of FOD preprocessing, hyperspectral monitoring models for winter wheat ChD were constructed using various machine learning algorithms based on the full spectrum band and the sensitive band screened by CARS, achieving rapid monitoring of winter wheat ChD.

## 2 Materials and methods

### 2.1 Experimental design

This study conducted a two-year winter wheat irrigation experiment from October 2020 to July 2022 at the Agricultural Station of Shanxi Agricultural University in Taigu District, Jinzhong City, Shanxi Province. The tested variety was ‘Jintai 182’, which was sown in October every year and harvested in July the next year. The average organic matter content of the tested soil was 13.92 g·kg^-1^, total nitrogen content was 1.19 g·kg^-1^, available phosphorus content was 17.43 g·kg^-1^, and available potassium content was 189.21 g·kg^-1^. Treatment started from the jointing stage of winter wheat, chose the jointing, flowering, and filling stages for irrigation. A total of 5 treatments were set up: T1(No irrigation), T2(Irrigation once during the jointing stage), T3(Irrigation once each during the jointing and flowering stage), T4(Irrigation once each during the jointing and filling stage), and T5(Irrigation once each during the jointing, flowering, and filling stage). Each irrigation amount was 60 mm, and each treatment was repeated 3 times. The experimental site constructed a water tank according to FAO standards, with a total of 15 plots, the area of each plot was 6 m^2^(2 m×3 m). Nitrogen, phosphorus, and potassium fertilizers were used as base fertilizers and applied uniformly before sowing. The fertilization standards were N: 150 kg·hm^-2^, P_2_O_5_: 120 kg·hm^-2^, and K_2_O: 120 kg·hm^-2^, respectively. The spacing between sowing rows was 20 cm. The remaining field operations were consistent with local farmers.

### 2.2 Index measurement

Canopy hyperspectral reflectance data and ChD of winter wheat were obtained at regreening, jointing, booting, heading, flowering, early grain filling, later grain filling, and maturity stages, a total of 8 stages of data were obtained.

The canopy hyperspectral data was measured using a Field-Spec 3.0 spectrometer(ASD Company, Boulder, CO, USA), with a collection wavelength range of 350-2500 nm. The 350~1000 nm spectral sampling interval is 1.4 nm, and the spectral resolution is 3 nm; the 1000~2500 nm spectral sampling interval is 2 nm, and the spectral resolution is 10 nm. In order to reduce the error caused by light changes, the spectral collection should be conducted in sunny and cloudless weather, with no wind or wind speeds below level 3, the collection time was arranged at 10:00-14:00. When measuring, place the probe 1 meter above the canopy and measure 10 times in each plot. A whiteboard was used to correct before each measurement. After removing the abnormal curve, the average spectrum was calculated as the final spectrum of that plot.

Several representative functional leaves(Before the jointing stage was first unfolded leaf, after the jointing stage was flag leaf) were collected at the location where canopy hyperspectral data was collected. The leaves were transferred to the room. After the veins were removed, the leaves were cut into pieces. 0.0500 g was weighed and put into a 25 ml volumetric flask. 96% ethanol was used to fix the volume to the scale line. The volumetric flask was stored in the dark and away from light. The volumetric flask was shaken for 3 to 4 times in the middle. After 24 hours, the absorbance values at 649 nm and 665 nm were measured by spectrophotometer. The concentration formula of chlorophyll a, b, and total chlorophyll was as follows:


$$C_{a}=13.95\times A_{665}-6.88\times A_{649}$$

$$C_{b}=24.96\times A_{649}-7.32\times A_{665}$$

$$C=C_{a}+C_{b}$$

Where, C_a_, C_b_, and C were the concentration of chlorophyll a, chlorophyll b, and total chlorophyll in the soaking solution (mg·L^-1^). A_649_ and A_665_ were the absorbance values measured at the wavelength of 649 nm and 665 nm respectively.

The calculation formula of chlorophyll content and ChD was:


$$\text{Chlorophyll content}(mg\cdot g^{-1}\;FW)=\frac{C\times V}{FW\times 1000}\times n$$

$$\text{CHD}(\text{g}\cdot {\text{m}}^{-2})=\text{Chlorophyll content}\times \text{Aboveground fresh leaf biomass}\times 1000$$

Where, V was the volume of soaking solution, which was 25 ml in this study; FW was the blade weight; N was the dilution ratio; 1000 was the unit conversion factor; The aboveground fresh leaf biomass refers to the fresh weight of all leaves per unit surface area (kg·m^-2^).

### 2.3 Data analysis methods

Before data analysis, the data set was divided into calibration set and validation set according to the 2:1 ratio using the concentration gradient method. Specifically, arranged the dependent variables from small to large, selected the first and third samples from every three samples to enter the calibration set, and the second sample to enter the validation set. At the same time, ensure that the maximum and minimum values of the data set were put into the calibration set.

#### 2.3.1 Fractional-order derivative

FOD was first proposed by Italian mathematician Samuelson in 1695 and had a history of over 300 years. However, systematic research on FOD has mainly focused on the past few decades (MaChado et al., 2011; Li et al., 2017). After years of development, many forms of FOD definitions have emerged, among which GL, RL, and Caputo are the most widely used definitions (Benkhettou et al., 2014; Yang et al., 2022b). The as follows:


$$\text{GL}:\frac{d^{\alpha }f\left(\lambda \right)}{d\lambda^{\alpha }}=\underset{h\to 0}{\lim}\frac{1}{h^{\alpha }}\sum_{m=0}^{\frac{p-q}{h}}{\left(-1\right)}^{m}\frac{\Gamma \left(\alpha +1\right)}{m!\Gamma \left(\alpha -m+1\right)}f\left(\lambda -mh\right)$$

$$\text{RL}:\frac{d^{\alpha }f\left(\lambda \right)}{d\lambda^{\alpha }}=\frac{1}{\Gamma \left(k-\alpha \right)}\frac{d^{k}}{dx^{k}}\int_{q}^{\lambda }\frac{f\left(\tau \right)}{{\left(\lambda -\tau \right)}^{\alpha -k+1}}d\tau ,\;0\leq \text{k}-1<\alpha <\text{k}$$

$$\text{Caputo}:\frac{d^{\alpha }f\left(\lambda \right)}{d\lambda^{\alpha }}=\frac{1}{\Gamma \left(k-\alpha \right)}\int_{q}^{\lambda }\frac{f^{\left(k\right)}\left(\tau \right)}{{\left(\lambda -\tau \right)}^{\alpha -k+1}}d\tau ,\;0\leq \text{k}-1<\alpha <\text{k}$$

Among them, α was a real number, representing any order, in this study, its value range was [0,2], with a step size of 0.1; λ and τ were wavelengths; For the GL method, f(λ) was the spectral reflectance value at λ wavelength, for the RL method and Caputo method, f(λ) was the spectral curve formula at λ; h was the differential step size, which was 1 in this paper; p was the upper limit of differentiation; q was the lower limit of differentiation; m represented the number of bands before the λ wavelength, and m=p-q; K was the smallest integer greater than α; ∑ was the summation symbol; Γ(x) was the Gamma function; ∫ was the integral symbol; f^(k)^(τ) represented taking the k-th derivative of function f(τ).

From the above equation, it can be seen that the GL method is based on a discrete definition. The FOD at point λ is related to the spectral reflectance of all wavelengths before that wavelength. For the convenience of calculation, combined with previous research, we uniformly selected the 40 points before λ for calculation (Yang et al., 2022b). Both the RL method and Caputo method need to first fit the formula f(λ) at λ. In this paper, the spectral reflectance at three consecutive wavelengths (i.e. λ-1, λ, λ+1) was selected for binomial fitting calculation of f (λ).

#### 2.3.2 Competitive adaptive reweighted sampling

CARS is an algorithm based on the regression coefficients of the PLSR model. The main idea of this algorithm is to mimic the “survival of the fittest” principle in Darwin’s evolutionary theory. Each time, a certain number of samples (usually 80% of all sample sizes) were selected through Monte Carlo sampling to construct the PLSR model, and the bands with the higher absolute weight of regression coefficients were selected as the new subset. Then selected samples through Monte Carlo sampling to construct the model. After multiple calculations, only two bands were retained to enter the model, and the cycle ended. From multiple PLSR models obtained, selected the band in the subset with the smallest root mean square error of cross-validation as the final selected feature band (Tang et al., 2023; Zhang et al., 2024).

#### 2.3.3 Modeling algorithm

PLSR is a modeling method that combines principal component analysis, canonical correlation analysis, and multiple linear regression analysis. It is currently one of the most effective linear regression methods in constructing hyperspectral models (Yang et al., 2022b). The other seven algorithms are all nonlinear regression methods. SVR mainly maps raw data to a high-dimensional feature space, and achieves regression tasks by finding a suitable hyperplane (Yang et al., 2022a). MLPR is a type of artificial neural network, which is a relatively simple neural network (Yang et al., 2022a). RFR is an ensemble algorithm based on DTR, which completes regression tasks by integrating the prediction results of multiple decision trees (Han et al., 2022). ETsR is an improved algorithm of RFR, which is an ensemble learning algorithm that reduces fitting errors by combining the prediction results of multiple extreme random trees (Han et al., 2022). DTR is a method of regression analysis of data by constructing a decision tree model (Arjmandi et al., 2023). KNR is an instance-based learning method that finds K nearest neighbor samples in a sample and performs regression prediction based on their labels (Maraden et al., 2023). GPR is a non-parametric model that uses Gaussian processes as priors and is a probability-based machine learning algorithm (Sahoo et al., 2023).

In this paper, Microsoft Excel 2021 was used to organize data. MATLAB 2021 was used to perform the FOD algorithm. Python 3.11 was used to perform CARS and modeling algorithms. Origin 2021 was used for mapping. Evaluate the accuracy of the model using the coefficient of determination (R^2^), root mean square error (RMSE), and relative predictive deviation (RPD).

## 3 Result and analysis

### 3.1 Descriptive statistical analysis

**Figure 1:**
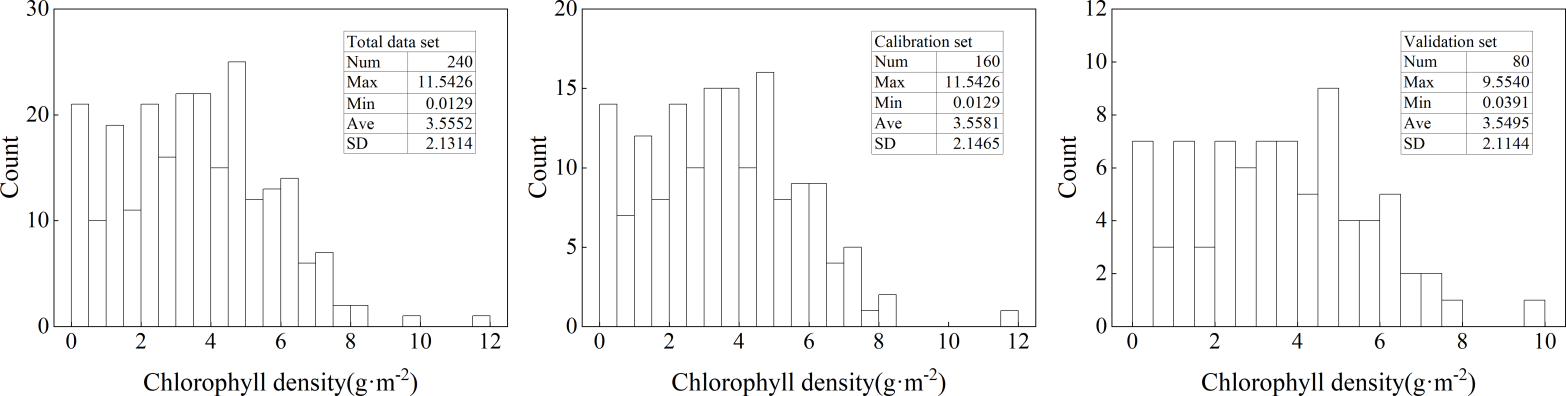
Descriptive statistical analysis of ChD. Num, Max, Min, Ave, and SD represent the number of samples, maximum, minimum, average, and standard deviation of the dataset, respectively. The same below.

**Figure 2:**
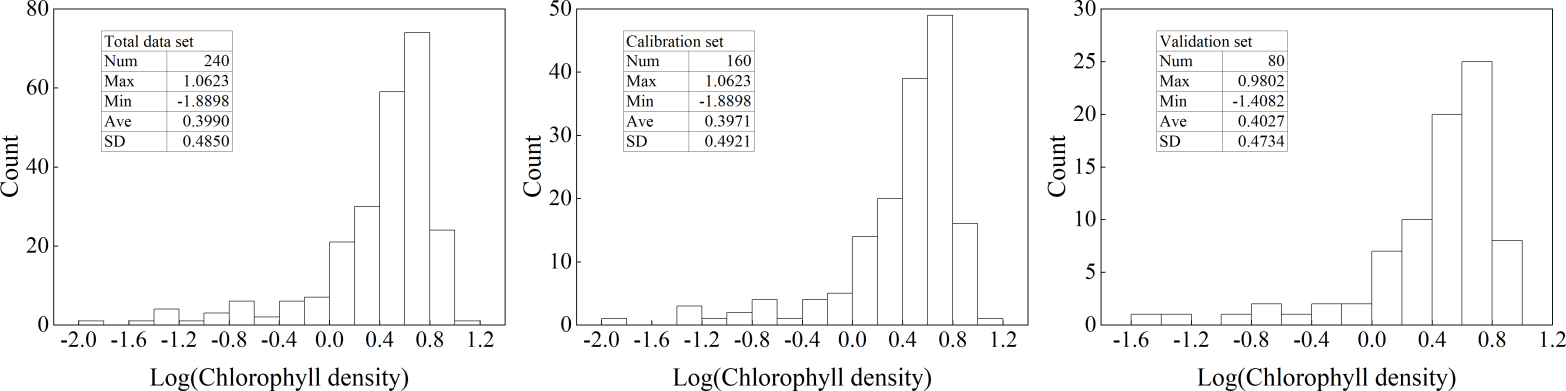
Descriptive statistical analysis of Log(ChD).

The descriptive statistical analysis results were shown in **Figure 1**. From the figure, it can be seen that the maximum and minimum values of the total data set were 11.5426 g·m^-2^ and 0.0129 g·m^-2^, respectively. When dividing the dataset, both the maximum and minimum values were assigned to the calibration set. The average value of the total data set was 3.5552 g·m^-2^, and the average values of the calibration and validation sets were also close to this. For the standard deviation, it showed that the total data set and calibration set were slightly higher than the validation set. However, the sample distribution of the three data sets does not conform to a normal distribution to a certain extent. Based on this, this study performed logarithmic processing on ChD data, and the results were shown in **Figure 2**. From the figure, it can be seen that, similar to **Figure 1**, the maximum and minimum values were also assigned to the calibration set when dividing the data set. The average and standard deviation of the three datasets were relatively close. However, after logarithmic processing, the distribution of the data sets was more in line with a normal distribution.

### 3.2 Changes in spectral reflectance

#### 3.2.1 Changes in original spectral reflectance

**Figure 3:**
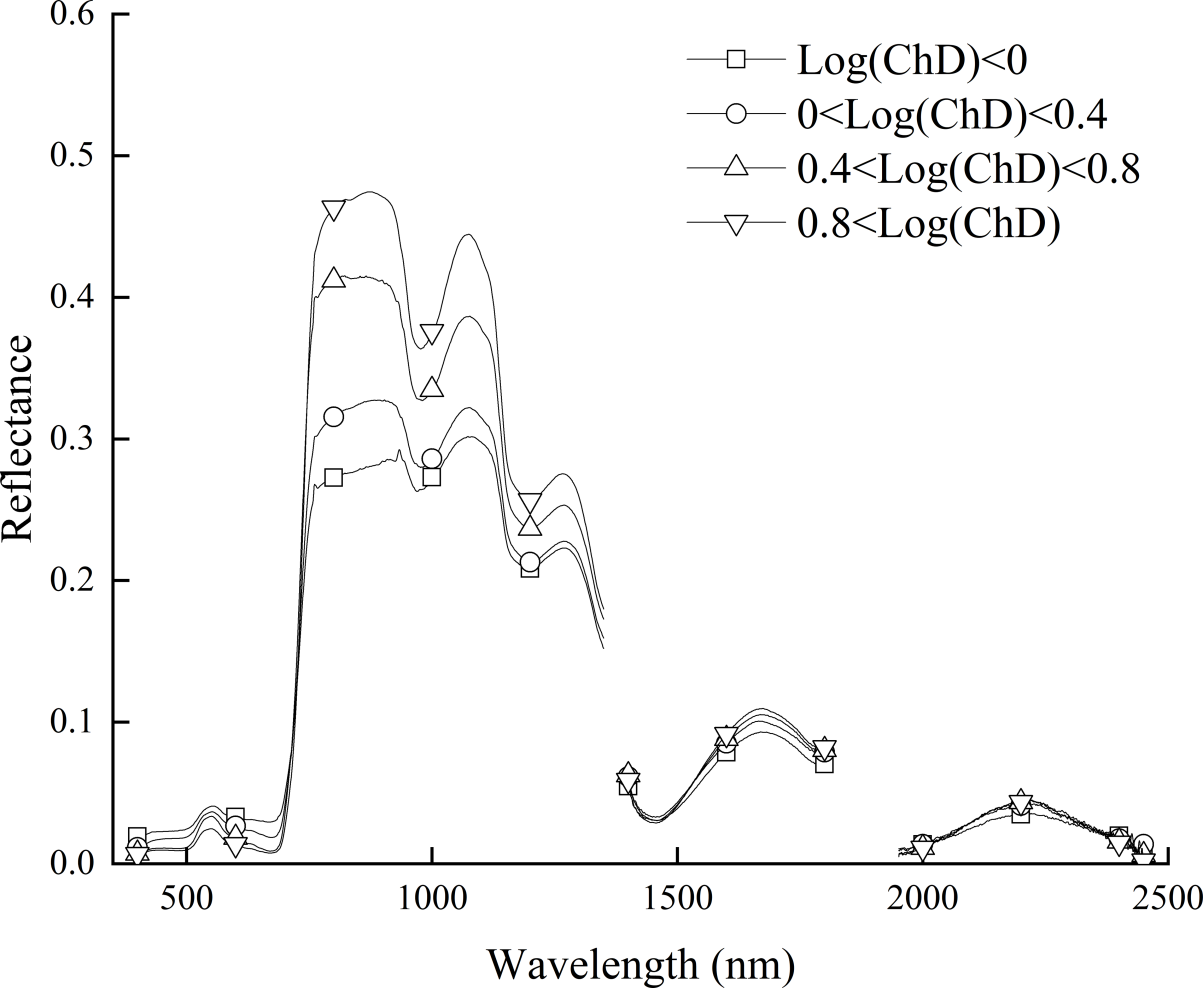
Changes in original spectral reflectance.

From **Figure 2**, it can be seen that the Log(ChD) values were mainly distributed in the range of 0 to 0.8. Therefore, this paper mainly analyzed the changes in the original spectral reflectance of samples with four gradients of Log(ChD)<0, 0<Log(ChD)<0.4, 0.4<Log(ChD)<0.8, and 0.8<Log(ChD), as shown in **Figure 3**. At the same time, in order to reduce the effect of factors such as moisture in the air, the bands within the range of 350-399 nm, 1351-1399 nm, 1801-1950 nm, and 2451-2500 nm in the original spectrum were removed. From the figure, it can be seen that the hyperspectral reflectance of winter wheat canopy mainly showed a trend of first increasing and then decreasing with the increasing of wavelength. In terms of details, a small reflection peak was formed near 550 nm, a near-infrared reflection platform was formed in the range of 780-1100 nm, and two obvious absorption valleys were formed near 1000 nm and 1450 nm, which was consistent with the basic characteristics of spectral reflectance of green plant canopies. In addition, in the range of 400-700 nm, samples with different Log(ChD) exhibited an overall trend of decreasing spectral reflectance with increasing Log(ChD), while in the range of 740-1800 nm, it showed an increasing trend with increasing Log(ChD). This indicated that there may be a certain quantitative relationship between Log(ChD) and the spectral reflectance at some specific wavelengths.

#### 3.2.2 The effect of FOD on spectral reflectance

**Figure 4:**
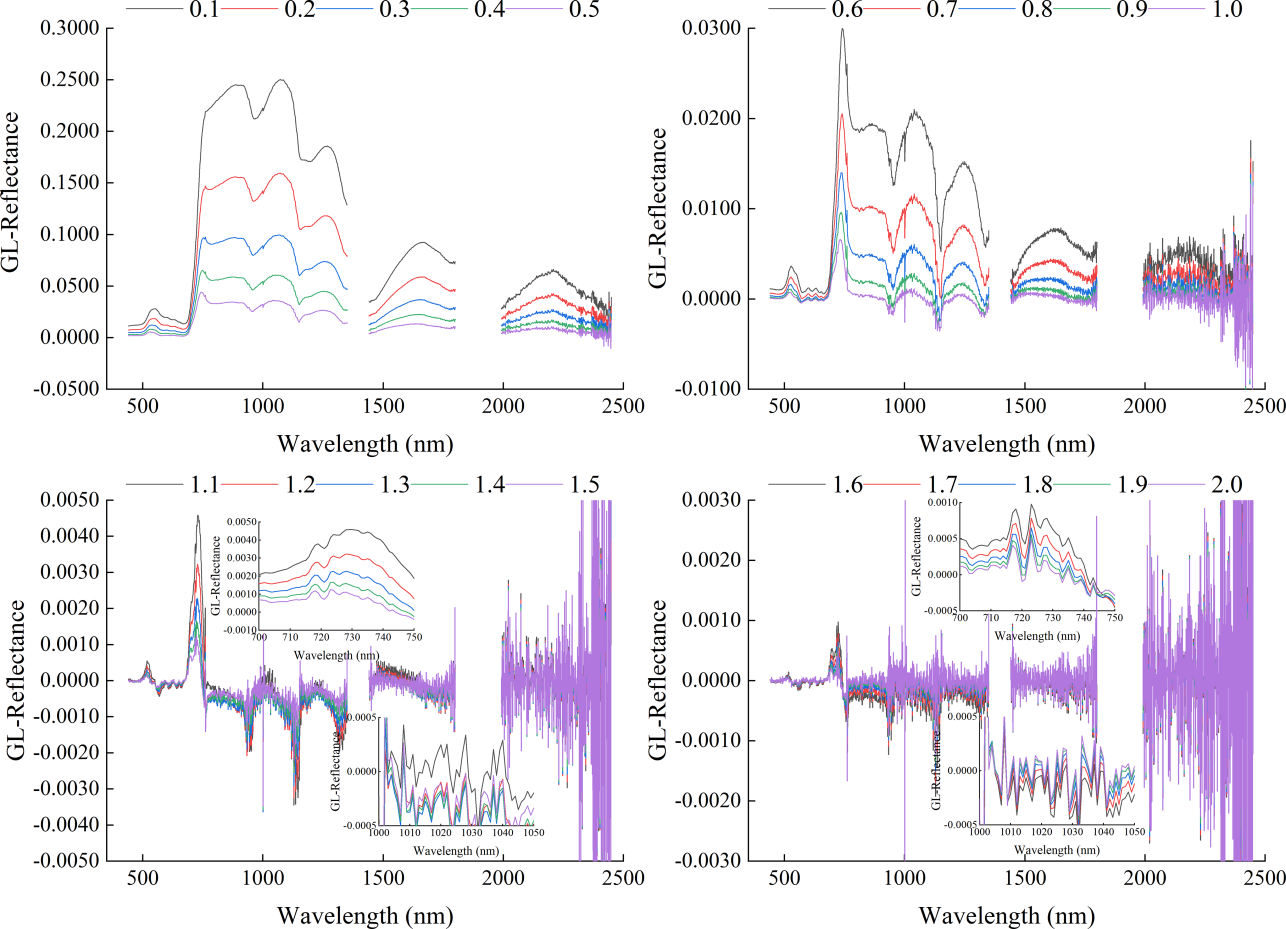
Spectral reflectance curve based on GL method preprocessing.

**Figure 4** showed the spectral reflectance curve based on GL-FOD preprocessing. Combined with **Figure 3**, it can be seen that as the order increased, there was a gradual change process in the GL spectral reflectance. In terms of details, there was a clear trend of gradual decreasing in the range of 0 to 1.0-order, and the smaller the order, the greater the magnitude of the decrease. In the range of 1.0 to 2.0-order, there were various trends of variation, such as a gradual decreasing in the visible light band range (such as 700-750 nm). In the partial band range of near-infrared (such as 1000-1050 nm), it generally showed a trend of first decreasing and then increasing, and reached its lowest point in the 1.4-order. In addition, compared to the change in spectral reflectance in the 0 to 1.0-order range, the change amplitude in spectral reflectance in the 1.0 to 2.0-order range was relatively small.

**Figure 5:**
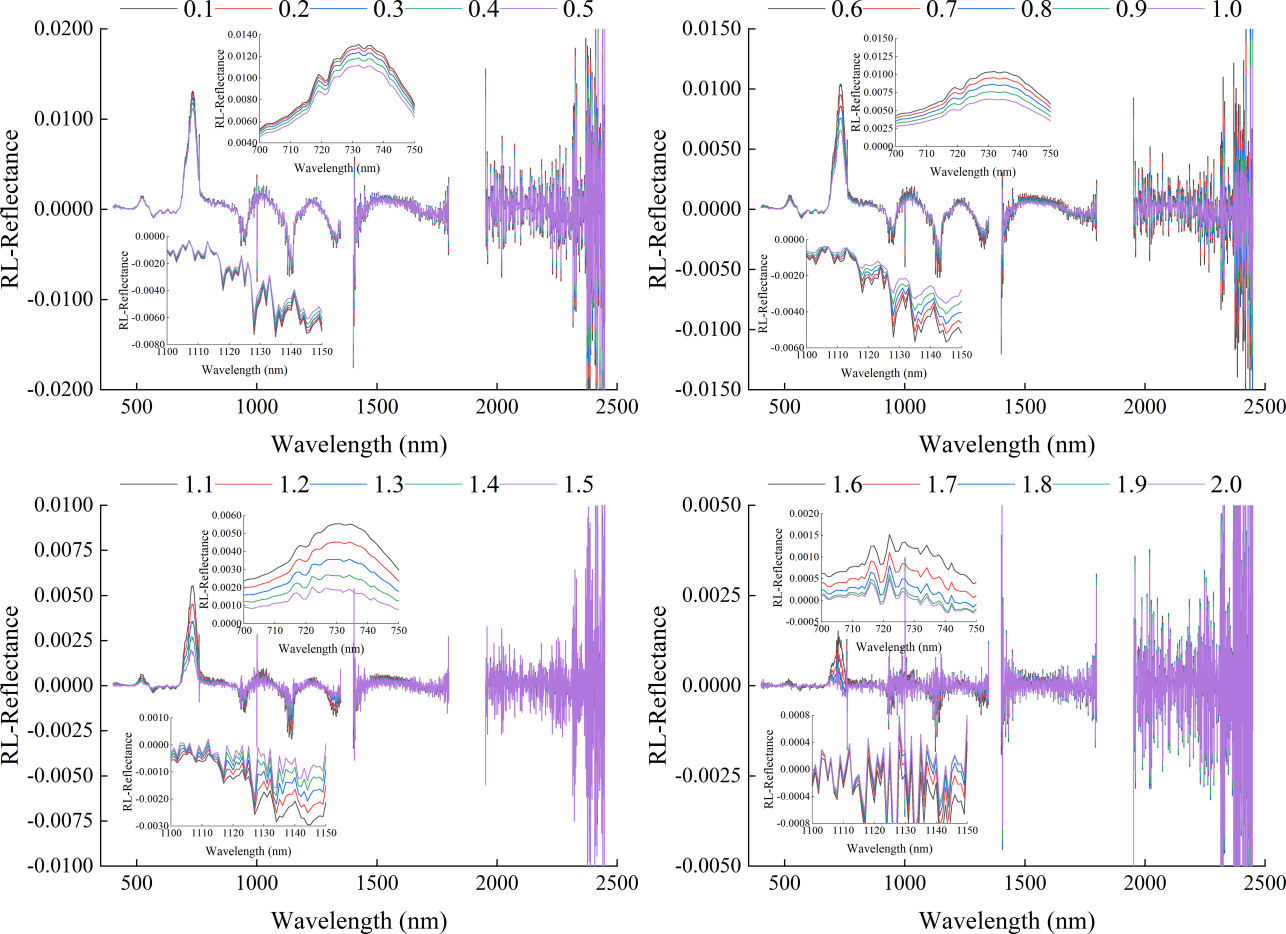
Spectral reflectance curve based on RL method preprocessing.

**Figure 5** showed the spectral reflectance curve based on RL-FOD preprocessing. Unlike the spectral curve changing trend after GL preprocessing, the 0.1-order RL-FOD spectral curve lost the basic characteristics of the original spectral curve and had a basic changing trend of the 1.0-order derivative. As the order increased, the overall change in RL spectral reflectance was also different from that of GL spectral reflectance. The main performance was that as the order increased, the RL spectral reflectance gradually increased and gradually decreased alternately. For example, in the range of 700-750 nm, it showed a gradually decreased trend, while in the range of 1100-1150 nm, it showed a gradually increased trend. And during the continuous 0.1-order variation process, the amplitude of the change in RL spectral reflectance was relatively small.

**Figure 6:**
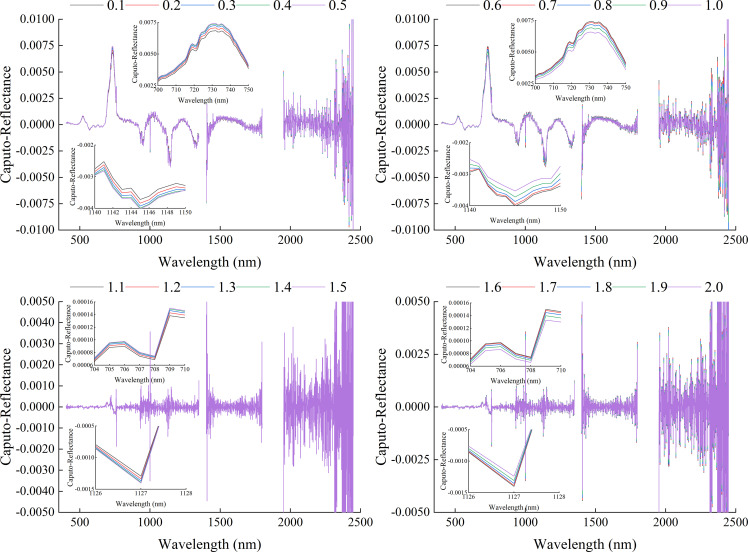
Spectral reflectance curve based on Caputo method preprocessing.

**Figure 6** showed the spectral reflectance curve based on Caputo-FOD preprocessing. Similar to the preprocessing results of the RL method, began from the 0.1-order, the Caputo-FOD spectral curve lost the basic characteristics of the original spectral curve. However, in the range of 0.1-1.0 order and 1.1-2.0 order, Caputo spectral reflectance exhibited similar changes with increasing wavelength. As the order changed, the Caputo spectral reflectance at the same wavelength alternated between increasing and decreasing. For example, in the range of 0.1 to 0.5-order, the spectral reflectance gradually increased in the range of 700 to 750 nm, while it gradually decreased in the range of 1140 to 1150 nm. The change in the range of 0.6 to 1.0-order was opposite to this, showing a gradual decreasing in the range of 700-750 nm and an increasing in the range of 1140-1150 nm. The performance in the range of 1.1 to 2.0-order was similar to that of 0.1 to 1.0-order, with 1.5-order and 1.6-order being the critical values for spectral reflectance changing at the same wavelength. However, within the range of 1.1 to 2.0-order, the changing amplitude of the Caputo spectral reflectance curve was smaller. It was worth noting that, unlike the GL and RL methods, the Caputo spectrum exhibited a significant changing amplitude from 1.0 to 1.1-order, and a gradual change cannot be observed, which may be related to different algorithmic properties.

### 3.3 Correlation analysis

**Figure 7:**
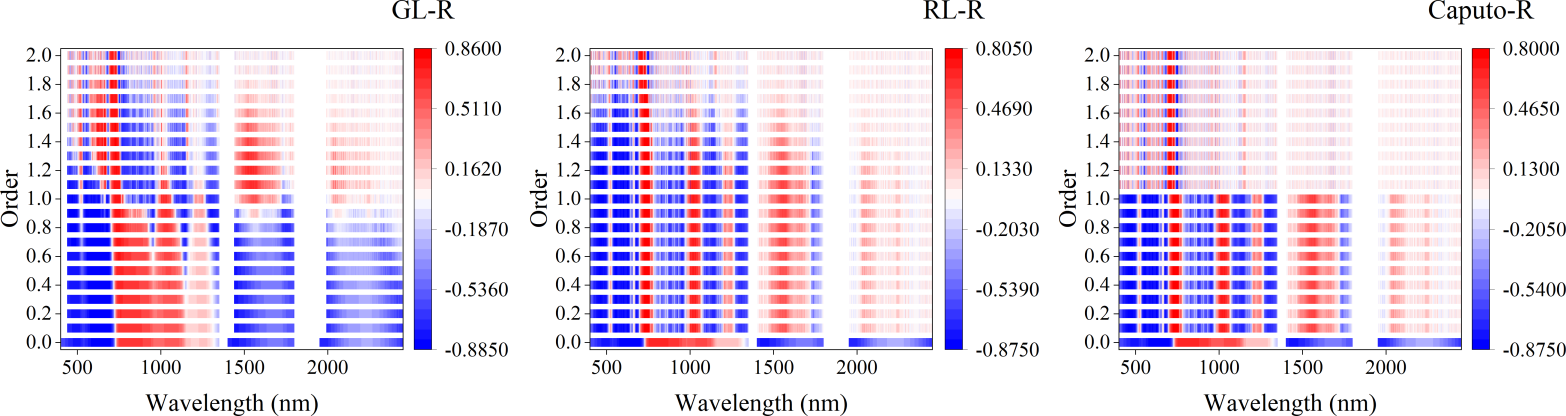
The correlation between different FOD spectra and Log(ChD). 0.0-order represents the original spectrum.

**Figure 7** showed the correlation coefficients between three FOD spectra and Log(ChD). From the figure, it can be seen that most of the bands in the original spectrum had a high correlation with Log(ChD), with a maximum correlation coefficient of -0.8625. The three types of FOD all increased the correlation between the original spectrum and Log(ChD) to varying degrees, but the correlation showed different laws of change. For GL-FOD, as the order increased, it can be clearly observed gradually changing that the correlation coefficient between the original spectrum to 2.0-order spectrum and Log(ChD), and followed the law of higher correlation with smaller order and shorter wavelength. For RL-FOD, only a gradual change in the correlation coefficient between the 0.1 to 2.0-order spectrum and Log(ChD) could be observed, and it also showed a law of higher correlation with smaller order and shorter wavelength. For Caputo-FOD, the correlation coefficients between the 0.1 to 1.0-order spectrum with the 1.1 to 2.0-order spectrum and Log(ChD) were the same basically. However, in the range of 0.1 to 1.0-order, the correlation between Caputo-FOD spectrum and Log(ChD) was higher, and the trend of the correlation coefficient between RL spectrum and Log(ChD) was similar.

### 3.4 CARS screened band results

**Figure 8:**
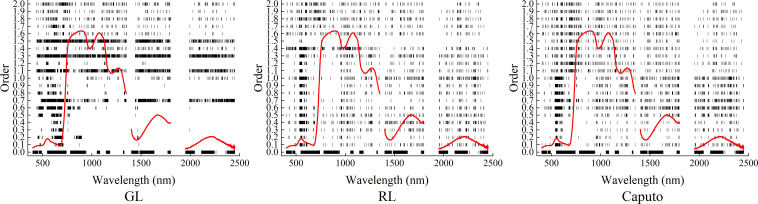
Position of bands screened by CARS based on three types of FOD spectra.

**Figure 8** showed the positions of bands screened by CARS based on three FOD spectra, and **Table 1** showed the number of bands screened by CARS. It can be seen that under different FOD spectra and order conditions, the minimum number of bands screened by CARS was 11 bands screened under 1.5-order RL-FOD spectral conditions, and the maximum was 1060 bands screened based on the original spectrum. Most of the number of screened bands were less than 200. In terms of the position of the selected bands, they were distributed throughout the entire spectral range. For the GL-FOD spectrum, the bands selected at lower orders were mainly concentrated in the visible light band range, with less distribution in the near-infrared band. The bands selected at higher orders were distributed throughout the entire spectral range. For RL-FOD and Caputo FOD spectra, the selected band positions were evenly distributed throughout the entire spectral range, but there was also a relatively concentrated distribution in the visible light band range.

**Table 1:** Number of bands screened by CARS based on three types of FOD spectra.

Order	GL	RL	Caputo	Order	GL	RL	Caputo
0.0	1060						
0.1	55	131	150	1.1	659	99	131
0.2	166	65	57	1.2	48	99	173
0.3	63	150	75	1.3	869	199	114
0.4	32	228	75	1.4	126	228	150
0.5	126	173	173	1.5	757	11	86
0.6	251	131	347	1.6	83	114	150
0.7	659	75	302	1.7	110	114	150
0.8	95	131	199	1.8	144	173	150
0.9	48	99	199	1.9	95	131	131
1.0	95	262	262	2.0	218	114	114

### 3.5 Hyperspectral monitoring model for ChD in winter wheat

#### 3.5.1 Hyperspectral monitoring model based on the full spectrum band

**Figure 9:**
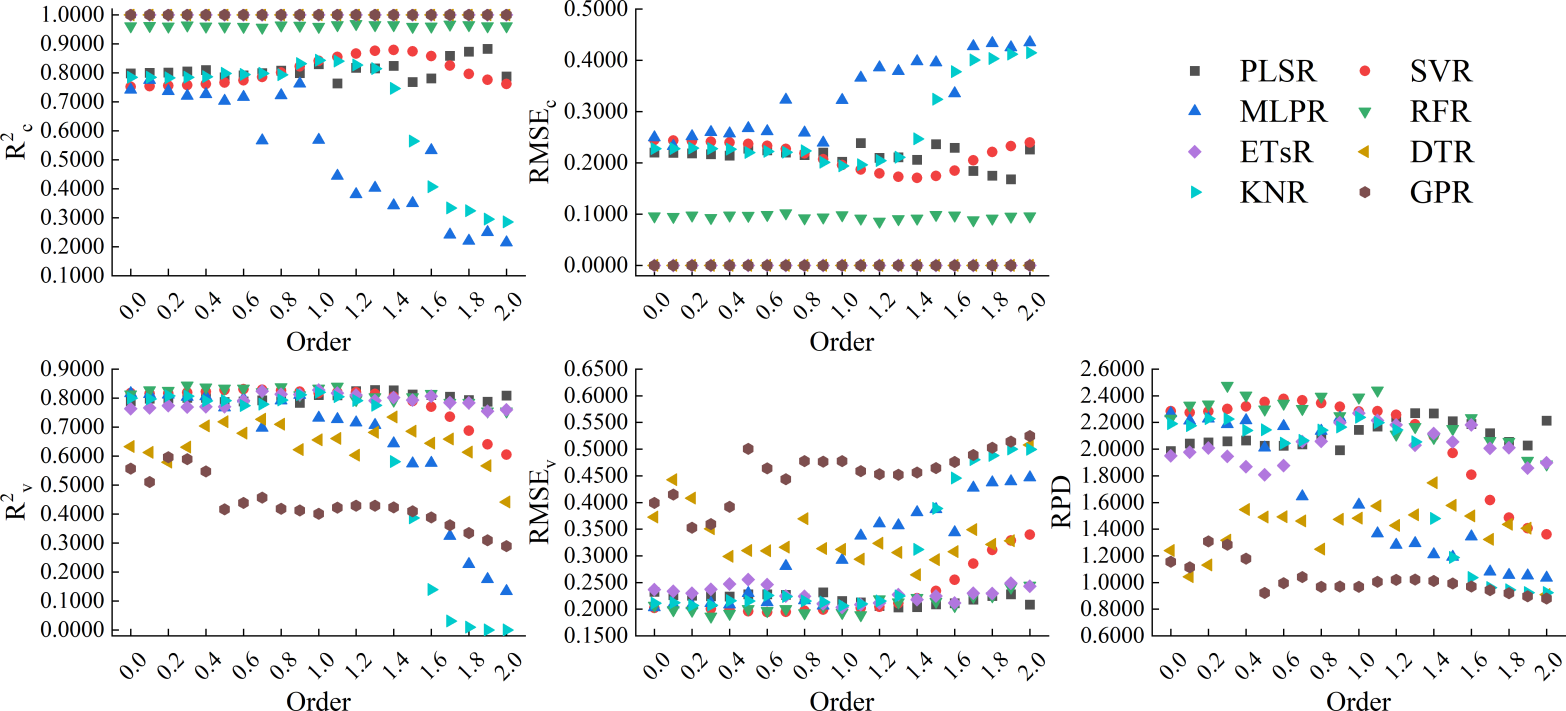
Modeling results based on the full spectrum band of GL-FOD spectra.

**Figure 9** showed the results of using 8 machine learning algorithms to construct models based on the full spectrum band of GL-FOD spectra. From the figure, it can be seen that in terms of modeling accuracy, all ETsR, DTR, and GPR models had high calibration accuracy, with R^2c^ of 1.0000 and RMSE_c_ of 0.0000. Next, was the model constructed based on RFR, whose R^2c^ was generally stable around 0.9600 and RMSE_c_ was stable around 0.0900. The model calibration accuracy based on PLSR and SVR was also high, with R^2c^ generally stable around 0.8000 and RMSE_c_ stable around 0.2000. The model constructed based on MLPR and KNR only had high calibration accuracy when the order was low, and as the order increased, its calibration accuracy decreased to varying degrees. Unlike calibration accuracy, in models with ETsR, DTR, and GPR with R^2c^ of 1.0000, only ETsR had relatively stable validation accuracy, with R^2v^ stable around 0.8000, RMSE_v_ stable in the range of 0.2000 to 0.2500, and RPD stable in the range of 1.8000 to 2.2000. The validation accuracy of the DTR model varied greatly with the changing of order, with R^2v^ mainly distributed in the range of 0.6000 to 0.7000. However, the change amplitude of RPD was large, with a maximum of 1.7476 and a minimum of only 0.9091. The validation accuracy of the GPR model was relatively low, and it gradually decreased with increasing order, with lower R^2v^ and RPD. The model constructed based on RFR and PLSR still had relatively stable validation accuracy, with R^2v^ stable around 0.8000 and RMSE_v_ stable around 0.2000. For the same order, the RPD of the RFR model was higher when the order was small, and the RPD of the PLSR model was higher when the order was high. For the SVR model, when the order was between 0.0-1.3, the model still had high validation accuracy, but as the order increased, the validation accuracy gradually decreased. Similar to calibration accuracy, models constructed based on MLPR and KNR also had high validation accuracy at lower order. Through comprehensive comparison, the model constructed by RFR based on the 0.3-order GL-FOD spectrum was the best among all models, with R^2c^, RMSE_c_, R^2v^, RMSE_v_, and RPD of 0.9640, 0.0931, 0.8442, 0.1865, and 2.4762, respectively. It had high accuracy and stability.

**Figure 10:**
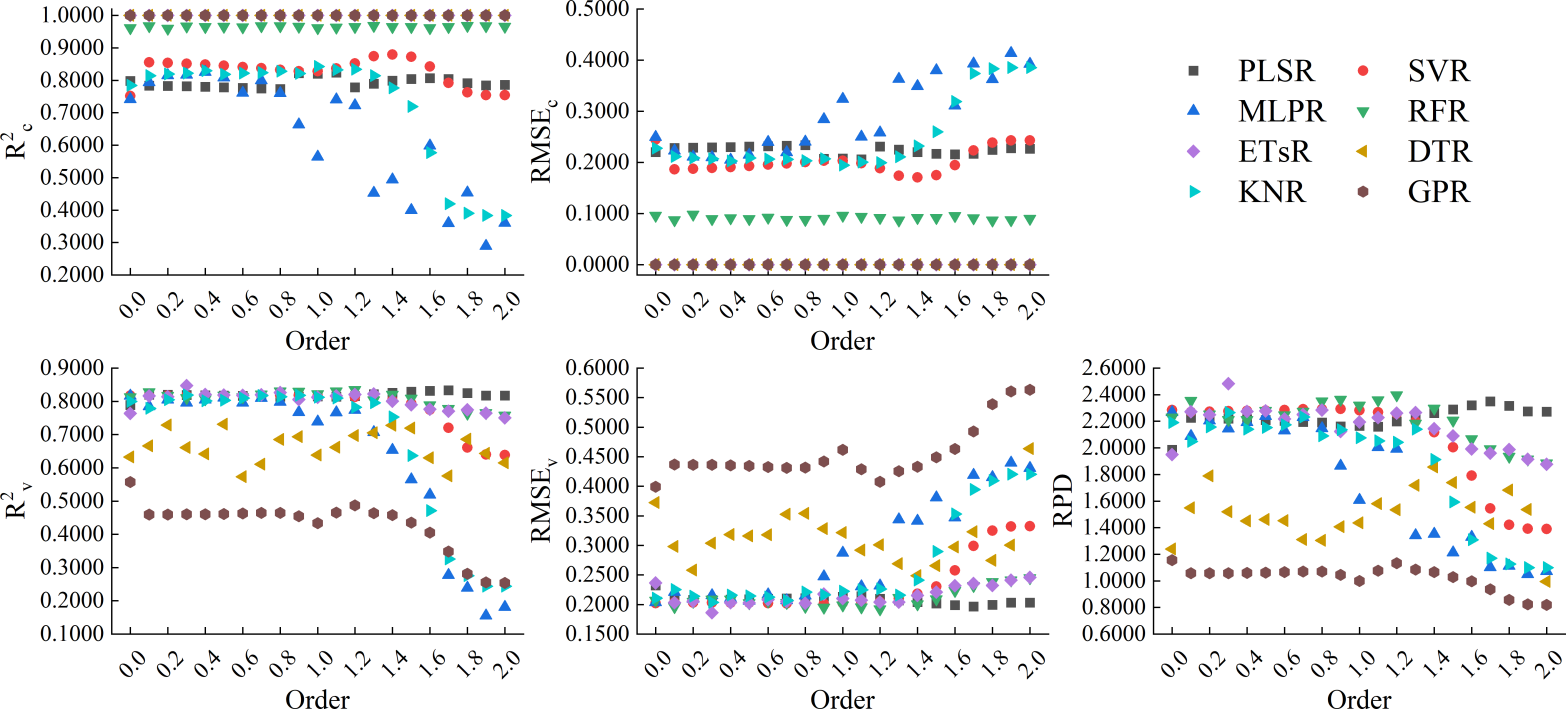
Modeling results based on the full spectrum band of RL-FOD spectra.

**Figure 10** showed the results of using 8 machine learning algorithms to construct models based on the full spectrum band of RL-FOD spectra. From the figure, it can be seen that the changes in calibration accuracy and validation accuracy of the models constructed based on RL-FOD spectra were similar to those of the models constructed based on GL-FOD spectra. Among all models, the model constructed by ETsR based on the 0.3-order RL-FOD spectrum had the highest accuracy, with R^2c^, RMSE_c_, R^2v^, RMSE_v_, and RPD of 1.0000, 0.0000, 0.8470, 0.1860, and 2.4823, respectively.

**Figure 11:**
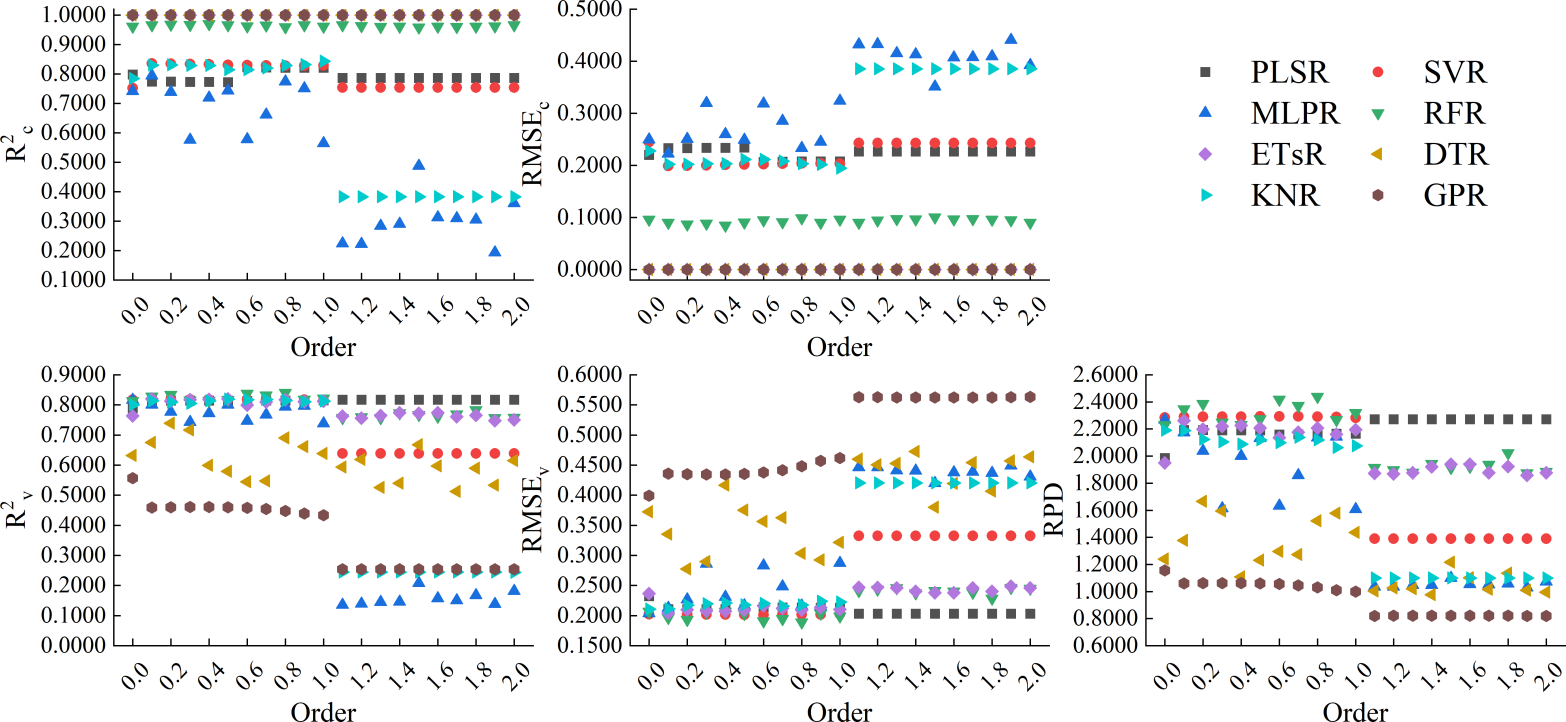
Modeling results based on the full spectrum band of Caputo-FOD spectra.

**Figure 11** showed the results of using 8 machine learning algorithms to construct models based on the full spectrum band of Caputo-FOD spectra. From the figure, it can be seen that similar to the GL and RL methods, the models constructed based on Caputo-FOD spectra by ETsR, DTR, GPR, and RFR still had high calibration accuracy, but only the ETsR and RFR models had relatively stable validation accuracy. The R^2c^ of the model constructed based on PLSR and SVR was stable around 0.8000, but the validation accuracy of the SVR model constructed in the range of 1.1 to 2.0-order was significantly reduced compared to the model constructed in the range of 0.1 to 1.0-order. The model constructed based on MLPR and KNR only had relatively high calibration accuracy and validation accuracy in the range of 0.0 to 1.0-order. Through comprehensive comparison, among all models constructed based on the full spectrum band of Caputo-FOD spectra, the model constructed by RFR based on the 0.8-order Caputo-FOD spectrum had the best accuracy, with R^2c^, RMSE_c_, R^2v^, RMSE_v_, and RPD of 0.9594, 0.0989, 0.8408, 0.1892, and 2.4398, respectively.

By comparison, it can be seen that among the models constructed based on the full spectrum band of the three FOD spectra, the model constructed by ETsR based on the full spectrum band of the 0.3-order RL-FOD spectrum had the highest accuracy.

#### 3.5.2 Hyperspectral monitoring model based on screening band

**Figure 12:**
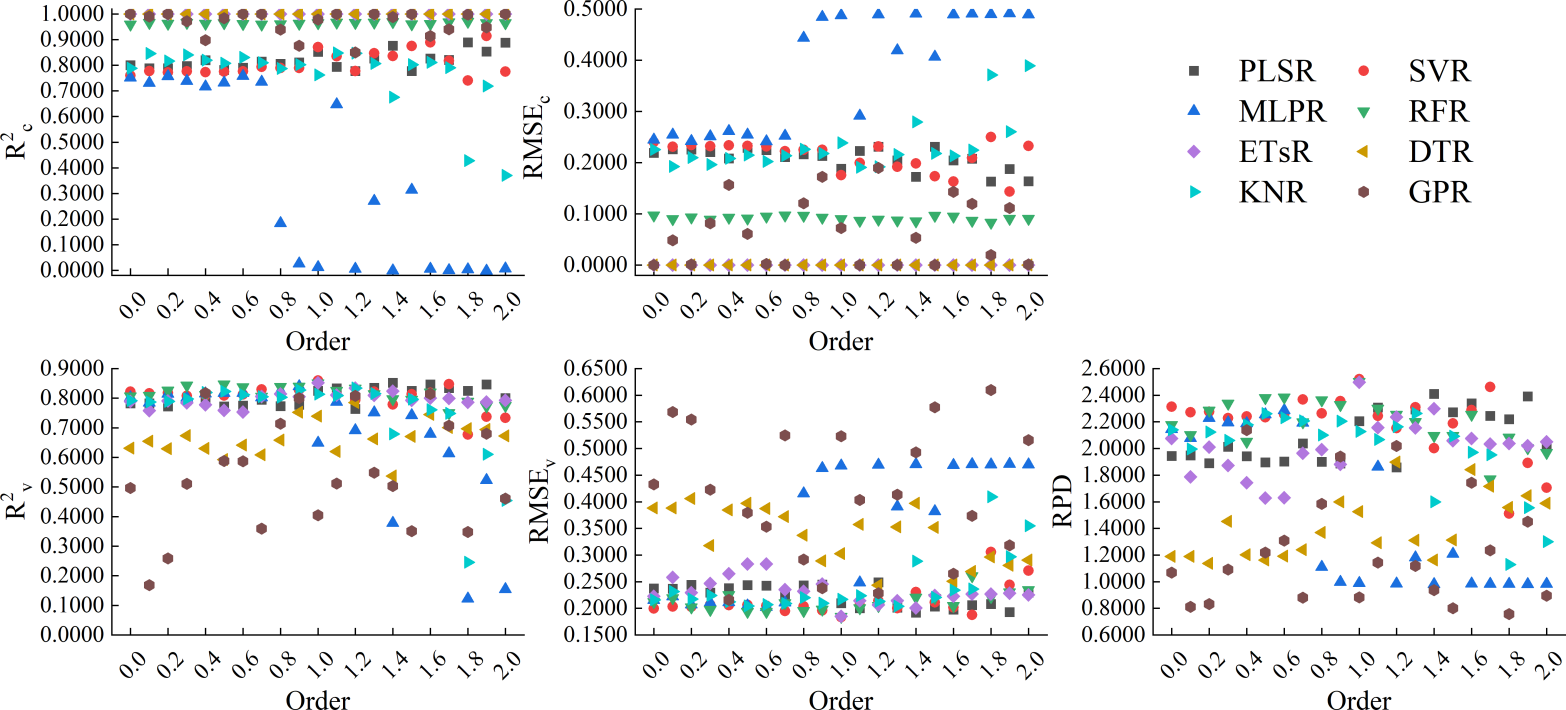
Modeling results based on the CARS band of GL-FOD spectra.

**Figure 12** showed the results of using 8 machine learning algorithms to construct models based on the CARS band of GL-FOD spectra. From the figure, it can be seen that the models constructed based on ETsR, DTR, GPR, and RFR all had high calibration accuracy, but only the models constructed based on ETsR and RFR had relatively high validation accuracy. The R^2c^ of the model constructed based on PLSR and SVR was stable around 0.8000, and the RMSE_c_ was distributed around 0.2000. For validation accuracy, the validation accuracy of PLSR model increased with increasing order, while the SVR model decreased with increasing order. The MLPR and KNR models only had high calibration and validation accuracy when the order was low, and the KNR model generally had higher accuracy than the MLPR model. Among all models, the model constructed by SVR based on the 1.0-order GL-FOD spectrum had the best accuracy, with R^2c^, RMSE_c_, R^2v^, RMSE_v_, and RPD of 0.8716, 0.1758, 0.8595, 0.1832, and 2.5205, respectively.

**Figure 13:**
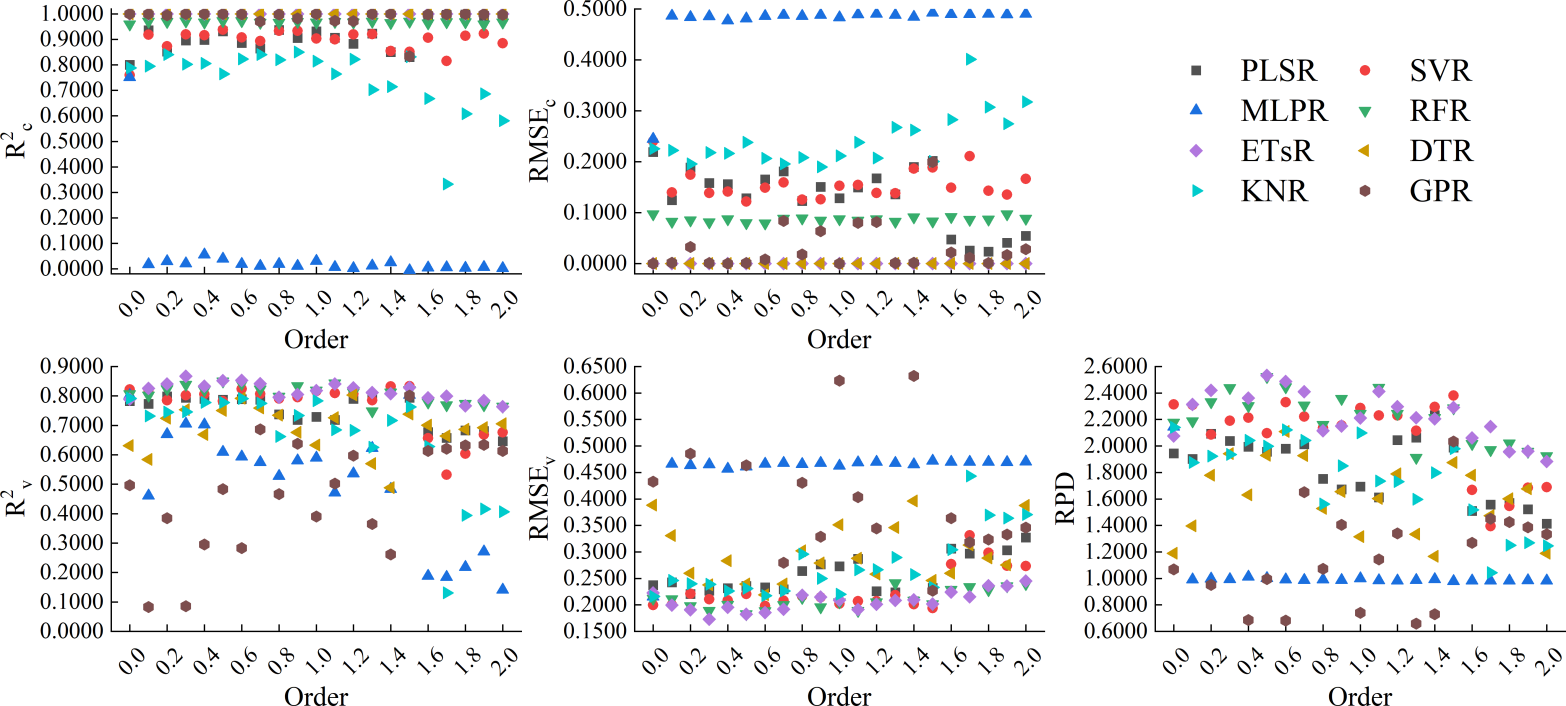
Modeling results based on the CARS band of RL-FOD spectra.

**Figure 13** showed the results of using 8 machine learning algorithms to construct models based on the CARS band of RL-FOD spectra. From the figure, it can be seen that the calibration accuracy of the models constructed based on ETsR, DTR, GPR, RFR, PLSR, and SVR was relatively high, with R^2c^ distributed in the range of 0.8000 to 1.0000 and RMSE_c_ distributed in the range of 0.0000 to 0.2000, respectively. The calibration accuracy of the model constructed based on KNR was relatively low, with R^2c^ mainly distributed between 0.6000 and 0.8500, and RMSE_c_ mainly distributed between 0.1900 and 0.3200. The calibration accuracy of models constructed based on MLPR was all relatively low, with R^2c^ approaching 0. Only models constructed based on ETsR and RFR had relatively high and stable validation accuracy in the validation model. Among all models, the model constructed by ETsR based on the 0.3-order RL-FOD spectrum had the best accuracy, with R^2c^, RMSE_c_, R^2v^, RMSE_v_, and RPD of 1.0000, 0.0000, 0.8667, 0.1732, and 2.6660, respectively.

**Figure 14:**
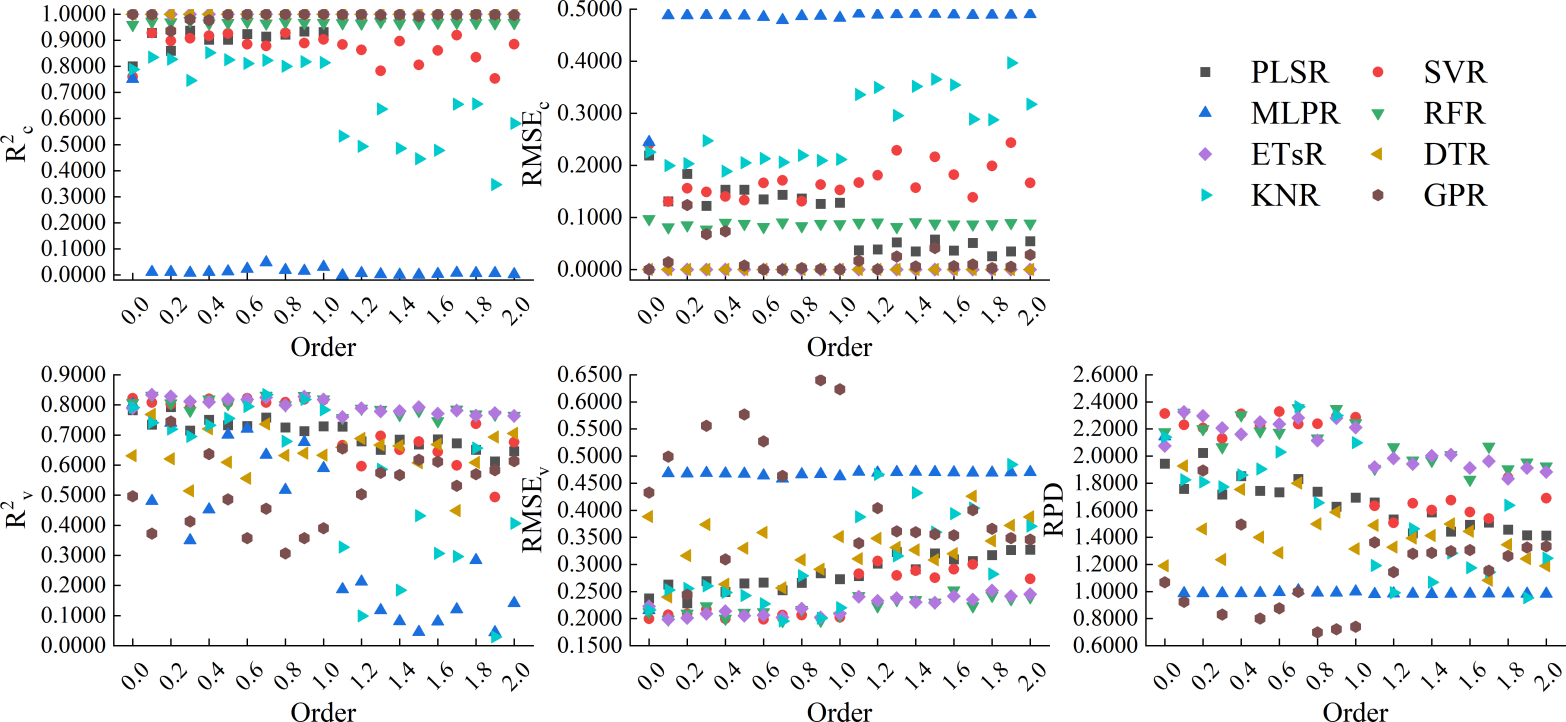
Modeling results based on the CARS band of Caputo-FOD spectra.

**Figure 14** showed the results of using 8 machine learning algorithms to construct models based on the CARS band of Caputo-FOD spectra. Similar to RL-FOD spectra, models constructed based on ETsR, DTR, GPR, RFR, PLSR, and SVR all had high calibration accuracy. Models constructed based on KNR only had high calibration accuracy when the order was small, while models constructed based on MLPR had low calibration accuracy. For validation accuracy, models constructed based on ETsR and RFR all had high and stable validation accuracy. Excepted for the MLPR model, the validation accuracy of all other models varied significantly with the increasing in order. Among all models, the model constructed by KNR based on the 0.7-order Caputo-FOD spectrum had the best accuracy, with R^2c^, RMSE_c_, R^2v^, RMSE_v_, and RPD of 0.8234, 0.2062, 0.8345, 0.1954, and 2.3630, respectively. Comparing all models constructed based on the CARS band, the model constructed by ETsR based on the 0.3-order RL-FOD spectrum had the best accuracy. At the same time, this was also the model with the highest accuracy obtained in this study. **Supplementary Figure 1** showed the 1:1 fitting figure of measured values and predicted values for this model. It can be seen from the figure that the model had a good prediction effect and can realize the hyperspectral estimation of Log(ChD).

## 4 Discussion

Proper preprocessing of the original hyperspectral reflectance data to reduce noise interference and improve the final model accuracy is an important step in the construction process of hyperspectral monitoring models. By comparing some previous studies, it can be seen that 1-order and 2-order derivative preprocessing in derivative preprocessing are common and effective spectral preprocessing methods (Ma et al., 2022; Cozzolino et al., 2023). However, there are significant differences in the original spectrum, 1-order derivative spectrum, and 2-order derivative spectrum curves, and there are also certain differences in the modeling results (Yang et al., 2023a). The emergence of FOD provided the possibility for studying the process of changes from original spectrum to integer-order derivative spectrum. There are many calculation methods for FOD, among which GL, RL, and Caputo are the three most widely used calculation methods. From its calculation method, it can be seen that the GL method is defined based on discrete points, making it very suitable for processing hyperspectral reflectance data. It is also the most commonly used method for applying FOD to hyperspectral research at present (Geng et al., 2024; Liu et al., 2023). The RL method and Caputo method have a similar principle, both of which first perform function fitting on discrete points before calculating FOD. The difference is that the RL method first integrates the function and then takes the derivative, while the Caputo method takes the derivative first and then the integral. It can be seen that the three methods have different calculation principles. This paper compared the roles of three FOD calculation methods in constructing hyperspectral monitoring models for winter wheat ChD. Because the ChD data obtained in this study does not follow a normal distribution, logarithmic processing was performed on the ChD data to make it conform to a normal distribution before further analysis (Qi et al., 2024). The results indicated that there may be a certain quantitative relationship between Log(ChD) and spectral reflectance at specific wavelengths, which was consistent with the research results of Feng et al. (2023). From **Figures 4**-**6**, it can be seen that the spectral curves preprocessed by the three FOD calculation methods had different variation characteristics. The GL-FOD spectra gradually lost the basic characteristics of the original spectral curve as the order increased, and the trend of the original spectral curve towards integer-order derivative can be observed clearly, especially the trend towards 1.0-order derivative curve. Previous studies have also shown that GL-FOD can display the process of changes from the original spectra to integer-order spectra, and it was believed that these subtle changes may provide more spectral features, thereby improving the accuracy of the final model (Cheng et al., 2022; Yang et al., 2022b). Both RL-FOD and Caputo FOD spectra lost the basic characteristics of the original spectra at the 0.1-order, which may be due to the calculation methods of these two methods. Because integer-order derivatives occurred in the calculation process of these two methods, this may directly lead to spectral curves in the range of 0.1-0.9 and 1.1-1.9 having varying characteristics of 1.0 and 2.0-order spectral curves, respectively (Yu and Liang, 2023; Alshammari et al., 2024). However, the trend of gradually changing from 0.1-order spectra to 2.0-order spectra can still be observed. The difference was that the difference between Caputo-FOD spectra of adjacent orders was smaller. Meanwhile, compared to the other two algorithms, the spectral curves of Caputo-FOD were extremely close in the range of 0.1-1.0 orders and 1.1-2.0 orders, respectively. This indicated that the Caputo method may not be sensitive to explaining the changes between integer-order derivative spectra, and correlation analysis also confirmed this. The correlation between Caputo-FOD spectra and Log(ChD) at the same wavelength was consistent in the range of 0.1-1.0 and 1.1-2.0 orders, respectively. Both GL and RL methods can observe a gradual change in correlation with the order changed at the same wavelength, and the correlation between GL-FOD spectra and Log(ChD) was more sensitive to changes in order. Moreover, the GL method can observe a trend in the correlation between the original spectra towards FOD spectra and the log(ChD), while the RL method and Caputo method cannot. This was also related to the fact that the GL method can reflect the gradual change of the original spectra towards the integer-order derivative spectra.

With the development of remote sensing technology in recent years, hyperspectral remote sensing technology not only has more spectral information but also increases the redundancy of spectral information. Meanwhile, according to the Hughes phenomenon, the accuracy of hyperspectral monitoring models is closely related to the number of bands entering the model (Hughes, 1968). Therefore, selecting a certain number of spectral bands from the full spectrum band of hyperspectral data to construct models has become one of the important contents of current hyperspectral-related research (Yang et al., 2023b). This paper used CARS to screen the bands in the original spectrum and three FOD spectra. From the screening results, it can be seen that in addition to 1060 bands screened out from the original spectrum, GL-FOD screened out 32-869 bands to varying degrees, RL-FOD screened out 11-262 bands, and Caputo-FOD screened out 57-262 bands. Excluding most of the spectral bands provided the possibility to reduce model complexity and improve model accuracy. The screening effect was similar to the research results of Sudu et al. (2022) on maize SPAD. In addition, although the bands screened by CARS were distributed throughout the entire spectrum, there was a more concentrated distribution in the visible light section when the order was low. Correlation analysis also indicated that the visible light bands of low-order FOD spectra had a high correlation with Log(ChD). This may be due to the more important relationship between some bands of visible light and the winter wheat chlorophyll. Feng et al. (2013) believed that the 650 nm and 670 nm wavelengths were important bands for monitoring winter wheat chlorophyll. Yang et al. (2023c) believed that the hyperspectral reflectance at 549 nm and the 1.0-order derivative spectrum at 735 nm had a strong correlation with wheat chlorophyll. The spectral reflectance at 536 nm, 596 nm, 674 nm, and the 1.0-order derivative spectra at 756 nm and 778 nm can be used to estimate winter wheat chlorophyll content. This study screened out the bands at these positions to varying degrees.

Different model construction algorithms directly determine the structure of the model, so choosing the appropriate modeling algorithm to construct the model is an important step in improving model accuracy. By analyzing previous research, it can be seen that PLSR is a common and widely recognized linear modeling algorithm with good performance (Yang et al., 2022b). Meanwhile, comparing the effectiveness of different machine learning algorithms in constructing models is also one of the current hot topics in hyperspectral research (Graham Ram et al., 2023; Silva et al., 2024). Therefore, this study selected 8 modeling algorithms, including PLSR, SVR, MLPR, RFR, ETsR, DTR, KNR, and GPR, to construct hyperspectral monitoring models of winter wheat Log(ChD) based on the full spectrum band and CARS band of three FOD spectra. From the model constructed based on the full spectrum band, it can be seen that the calibration accuracy of the model was relatively high when using ETsR, DTR, GPR, and RFR to construct the model. This was because DTR can effectively handle outliers and noise interference, reducing their effect on model accuracy, ETsR and RFR were both ensemble learning algorithms based on multiple decision trees. While GPR can capture complex structures and nonlinear relationships in the data. Therefore, the accuracy of the models constructed by them was high, but it was still necessary to be cautious of overfitting phenomena (Kwon et al., 2022; Sahoo et al., 2023). Secondly, the calibration accuracy of PLSR and SVR models was also relatively stable. The calibration accuracy of models constructed by MLPR and KNR decreased with increasing order. In terms of validation accuracy, models constructed based on GL-FOD and RL-FOD spectra exhibited a similar law of validation accuracy. The model constructed based on ETsR, PLSR, SVR, and RFR had high and stable validation accuracy, while the validation accuracy of the other 4 algorithms fluctuated significantly with changes in order. The model constructed based on Caputo-FOD spectra mainly showed that a certain algorithm had a relatively close accuracy in the range of 0.1-1.0 orders and 1.1-2.0 orders, respectively. This may be related to the close reflectance of Caputo-FOD spectra at the same wavelength, further indicating that the Caputo method was not sensitive to changes in FOD spectral curves. In addition, some algorithms such as GPR and DTR showed varying degrees of overfitting among the 8 modeling algorithms. Previous studies have suggested that this may be related to the high complexity and excessive number of iterations of the model. In future research, overfitting can be reduced by adjusting the number of iterations or regularization methods (Nansen et al., 2013; Singh et al., 2022). Among all models constructed based on the full spectrum band, the model constructed by ETsR based on the 0.3-order RL-FOD spectrum had the highest accuracy, with R^2c^, RMSE_c_, R^2v^, RMSE_v_, and RPD of 1.0000, 0.0000, 0.8470, 0.1860, and 2.4823, respectively.

This paper also constructed hyperspectral monitoring models of winter wheat Log(ChD) based on the bands selected by CARS. The results showed that the model constructed by ETsR, PLSR, SVR, RFR, SVR, and PLSR all had varying degrees of high calibration accuracy, but the calibration accuracy of the KNR model fluctuated significantly with increasing order. When using MLRP to construct models based on RL-FOD and Caputo FOD spectra, the calibration accuracy was extremely low, indicating serious underfitting during model construction, which made the model unusable. This indicated that MLPR was not suitable for constructing Log(ChD) hyperspectral monitoring models under these conditions. Excepted for models constructed by ETsR and RFR, which had relatively stable validation accuracy, the validation accuracy of other models had significant fluctuations. Among all models constructed based on CARS band, the model constructed by ETsR based on the 0.3-order RL-FOD spectrum had the highest accuracy, with R^2c^, RMSE_c_, R^2v^, RMSE_v_, and RPD of 1.0000, 0.0000, 0.8667, 0.1732, and 2.6660, respectively.

Through comprehensive comparison, it can be seen that both models based on full spectrum band and models based on CARS band achieved the highest monitoring accuracy when calculating FOD spectra using the RL method. It can be seen that compared to GL and Caputo methods, the RL method was more suitable for preprocessing hyperspectral reflectance data to improve model accuracy. However, if it was necessary to observe the gradient process from the original spectrum to the integer-order derivative spectrum, the GL method needed to be used for calculation. Among all the models constructed in this study, the original spectral curve was preprocessed with 0.3-order RL-FOD, combined with CARS, the model constructed by ETsR was the most accurate and could achieve hyperspectral monitoring of Log(ChD). This model only used 150 bands, reducing the number of bands by over 90% compared to the full spectrum band, greatly reducing the complexity of the model. For CARS, its advantage was that the number of bands retained during each iteration can be adjusted by adjusting the number of iterations, so that an appropriate number of bands can be selected to construct a model as the number of bands gradually decreases. However, due to the randomness involved in its calculation process, it was still possible to screen out some bands that were not related to ChD. At the same time, the number of bands screened by this research was still large, which makes the model may still have a high cost in actual use, thus limiting the use of the model. Therefore, in subsequent research, it may be considered to use algorithms such as the random frog algorithm to perform secondary screening on the screening results, while ensuring the accuracy of the model and further simplifying it (Chen et al., 2023). In addition, in order to improve the reliability and portability of the model, the accuracy of the model should also be further validated through the combination of unmanned aerial vehicles and other platforms in the follow-up research, so as to realize the rapid estimation of winter wheat ChD more efficiently.

## 5 Conclusion

This study focused on winter wheat, measured its ChD and corresponding canopy hyperspectral reflectance. By calculating Log(ChD), three FOD calculation methods, GL, RL, and Caputo, were used to preprocess the original spectral data. Based on the full spectrum band and CARS band, 8 machine learning algorithms, PLSR, SVR, MLPR, RFR, ETsR, DTR, KNR, and GPR, were used to construct hyperspectral monitoring models of winter wheat Log(ChD). The main conclusions were as follows:

1. GL-FOD can be used to observe the gradual change from the original spectral curve to the integer-order derivative spectral curve, while RL-FOD and Caputo-FOD spectra both exhibited varying degrees of changes in integer-order derivative spectra, but Caputo-FOD spectra were less sensitive to changes in order compared to GL-FOD and RL-FOD spectra. All three types of FOD spectra can improve the correlation between the original spectral curve and Log(ChD) to varying degrees, but only GL-FOD and RL-FOD can observe the change process of the correlation between FOD spectra and Log(ChD) with changes in order, and it showed that the shorter the wavelength, the lower the order, and the higher the correlation.
2. The CARS method can remove most of the spectral band, and the screened bands were generally distributed throughout the entire spectral range, but had a relatively concentrated distribution in the visible light range.
3. The models constructed based on the full spectrum band and the CARS band all had the best accuracy and stability at using ETsR based on 0.3-order RL-FOD spectrum. Among the three FOD calculation methods, the RL method was more suitable for constructing hyperspectral monitoring models for winter wheat ChD. The CARS method can simplify the model while improving its accuracy. Among all models, the model based on the 0.3-order RL-FOD spectrum, using CARS to screen bands and combining with ETsR, had the highest accuracy. Its R^2c^, RMSE_c_, R^2v^, RMSE_v_, and RPD were 1.0000, 0.0000, 0.8667, 0.1732, and 2.6660, respectively, which can achieve hyperspectral monitoring of ChD in winter wheat.

## Data Availability

The data analyzed in this study is subject to the following licenses/restrictions: The data that has been used is confidential. Requests to access these datasets should be directed to Chenbo Yang, ycb232008@sxau.edu.cn.

## Supplementary material

SUPPLEMENTARY FIGURE S1 1:1 fitting figure of Log (ChD)
