# Supplementary figures and images for: Hyperspectral estimation of chlorophyll density in winter wheat using fractional-order derivative combined with machine learning
Source: Front Plant Sci. 2025 Jan 14;15:1492059. doi: 10.3389/fpls.2024.1492059 (PMC11772485; doi:10.3389/fpls.2024.1492059)

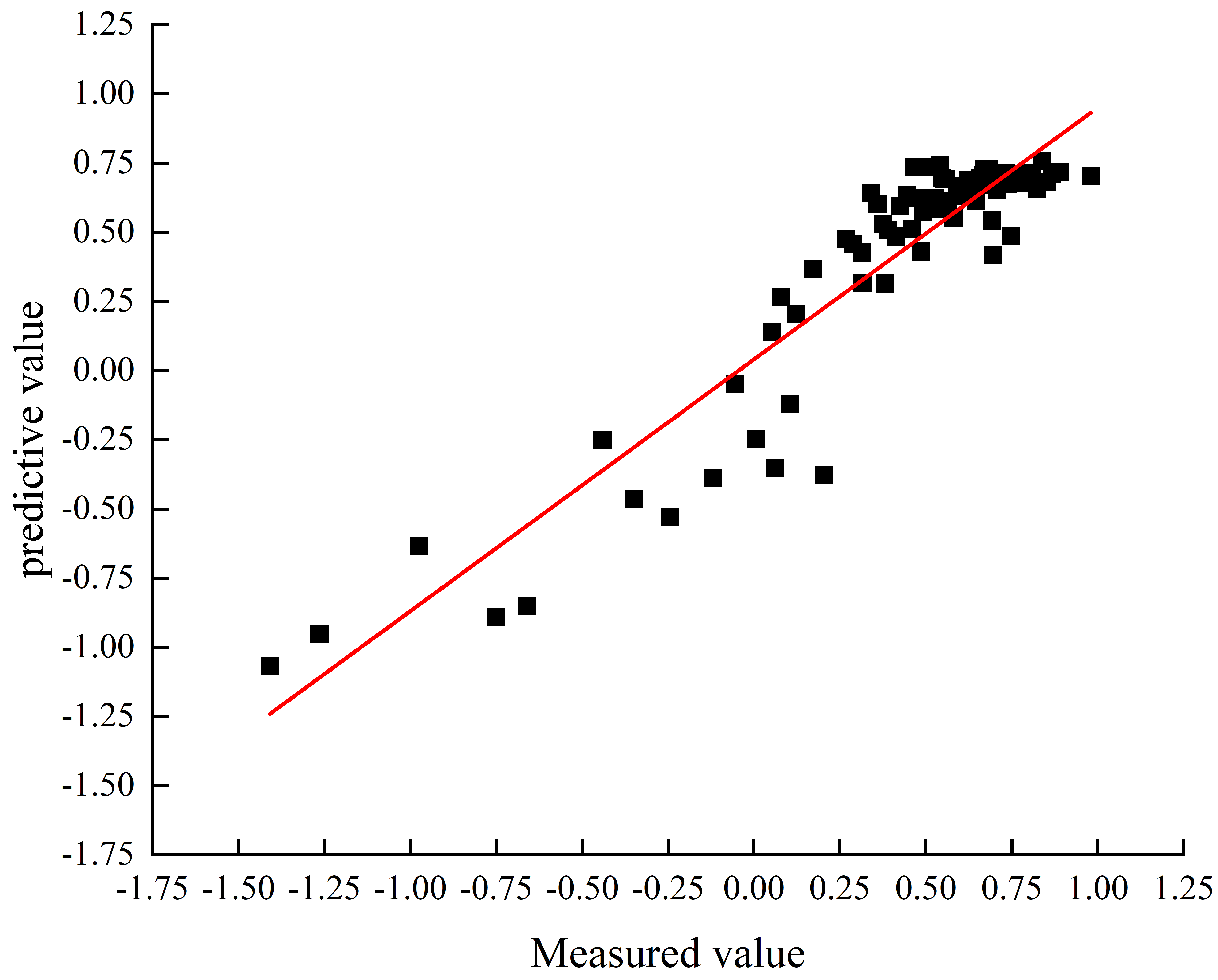

Supplement: SUPPLEMENTARY FIGURE S1 — 1:1 fitting figure of Log (ChD) [file Image1.tif]
